# Exploring and Expanding Supererogatory Acts: Beyond Duty for a Sustainable Future

**DOI:** 10.1007/s10551-022-05144-8

**Published:** 2022-06-24

**Authors:** Gareth R. T. White, Anthony Samuel, Robert J. Thomas

**Affiliations:** 1 South Wales Business School, University of South Wales, Pontypridd, CF37 1DL UK; 2 Senior Lecture Marketing and Strategy, Cardiff Business School, Cardiff University, Colum Road, Cardiff, CF10 3EU UK; 3 Lecturer Strategy and Marketing, Aston Business School, Aston University, Birmingham, B4 7ET UK

**Keywords:** Supererogation, Favour, Forgiveness, Forbearance, Sharing, Sustainability, Other-regarding

## Abstract

Supererogation has gained attention as a means of explaining the voluntary behaviours of individuals and organizations that are done for the benefit of others and which go above what is required of legislation and what may be expected by society. Whilst the emerging literature has made some significant headway in exploring supererogation as an ethical lens for the study of business there remain several important issues that require attention. These comprise, the lack of primary evidence upon which such examinations have been made, attention has been given to only singular pro-social acts of organizations, and the focus has been upon the actions of large organizations. Furthermore, Heyd’s (Supererogation, Cambridge University Press, 1982) original taxonomy of six supererogatory acts, comprising Moral Heroism, Beneficence, Volunteering, Favour, Forgiveness and Forbearance, has been considered to be complete and other forms of supererogatory acts have not yet been explored. In order to address these gaps this study poses the research questions: First, it studies how a single, contemporary SME performs multiple supererogatory acts in its attempts to address its social and environmental goals that go beyond CSR. Second, it seeks to gain a deeper theoretical understanding of Heyd’s (Supererogation, Cambridge University Press, 1982) taxonomy of six forms of supererogation through the capture of primary data. This research makes a three-year case study examination of a single SME that has been formally recognized for its work in addressing social and environmental issues at local, national and global levels. Primary data are acquired of the supererogatory acts that it performs through a three-year participant observation case study, utilizing 61 interviews and 3 focus groups with internal and external stakeholders. In doing so, it addresses the empirical limitations of the extant research, substantiates each of the forms that supererogatory acts may take, and makes a contribution to the theory of supererogation by identifying a further class of act that is ‘Sharing’.

## Introduction

There is a vast and growing literature that examines the behaviour of organizations toward society and the environment that comprise Corporate Social Responsibility (CSR). CSR is an evolving concept (Sarker & Searcy, 2016) and the boundaries of organizational responsibility are not universally agreed (Ohreen & Petry, 2012; Okoye, 2009). Much of this literature adopts Carrol’s (1979) original conceptualization of CSR and comprises the legislated expectations and limitations that are placed upon business, as well as those acts that go beyond what is enforced (McWilliams & Siegel, 2001) or what may be expected of a rational commercial enterprise (Margolis & Walsh, 2003). Whilst the need to reduce the deleterious effects of business activities has become enshrined in CSR frameworks and legislation, there is a growing recognition that the study and performance of this alone is insufficient to achieve triple-bottom-line (TBL) sustainability (Barnett et al., 2020; Fukuda & Ouchida, 2020; Hira, 2020; Mazutis, 2014; White et al., 2015).

A further criticism of CSR is its commercial dominance and marketing focus, and research has identified its relationship with unethical practices such as greenwashing (Gosselt et al., 2019), woke-washing (Vredenburg et al., 2020) and image laundering (Renard, 2003). This work has brought into question CSR’s ability to authentically capture and promote positive social, ecological and economic activity (Illia et al., 2013; Samuel et al., 2018). Subsequently it has been argued that voluntary, other-regarding and indeed many authentic pro-social actions of organizations cannot be readily explained by contemporary CSR business theory and require exploration with alternative ethical theories (Mazutis, 2014).

Supererogation (Heyd, 1982) has recently began to garner academic interest, with both Tencati et al. (2020) and Mazutis (2014) arguing it to be a suitable lens through which the non-mandated ‘good deeds’ of organizations are better understood and theorized. For instance, Tencati et al. (2020) use it to develop a qualified account of the ‘other-regarding’ acts of organizations that is “*superior to conventional CSR reasoning*” (p. 250) and Mazutis (2014) expands supererogation theory to develop a nuanced deontological framework of CSR to improve upon its “*conceptual clarity*” (p. 517). Whilst both papers advocate supererogation theory as a means of exploring and explaining the ethics of businesses, they also expose the dearth of work using this lens and set the foundations for a number of research opportunities to advance its utilization.

There are several notable deficiencies in the current supererogation literature. First, there is a lack of primary research to exemplify these types of acts in contemporary organizations. Both Mazutis (2014) and Tencati et al. (2020) draw upon accounts of these types of acts that have been reported in popular media. Second, research has tended to explore singular examples of the supererogatory behaviour of organizations. No research has yet attempted to identify organizations that perform multiple types of supererogatory acts. Third, the existing literature is dominated by examples of supererogatory acts that are performed by the large, multinational organisations, thus ignoring the role of SMEs in pioneering ethical business practices. Last, there have been no attempts to develop Heyd’s (1982) taxonomy of six types of supererogatory acts. Heyd (1982) acknowledged that his typology was not necessarily complete and that supererogatory acts may take other forms.

In response to these shortcomings, and to advance the application and understanding of supererogation, we immersed ourselves in the novel research landscape of Forest Green Rovers, an ethically proactive SME and ‘the world’s most sustainable football club’, in order to answer the following research questions:

First, it studies how a single, contemporary SME performs multiple supererogatory acts in its attempts to address its social and environmental goals that go beyond CSR.

Second, it seeks to gain a deeper theoretical understanding of Heyd’s (1982) taxonomy of six forms of supererogation through the capture of primary data.

The paper is structured as follows:

First, we review the Levinasian notion of ‘Others’ and the ‘Other-regarding’ actions that are a foundation of ethical business. Following this, we draw distinction between the various manifestations of pro-social behaviors that are characterized by their dependence upon reciprocity. This is followed by an examination of supererogation theory, the difference between the motivations and the intentions to perform supererogatory acts, and the type of acts that may be deemed supererogatory. We then review Mazutis’ (2014) and Tencati et al.’s (2020) seminal studies of supererogation. Next, the Research Context is described and the Methodological choices are discussed before the Findings and Discussion are presented. This section ends with a discussion of the primary contribution of the study that is an extension of the taxonomy of supererogatory acts to include the class of ‘Sharing’. The paper closes with statements of contributions, limitations and suggestions for future research.

### Ethical Business and ‘Others’

The study of business ethics frequently draws upon the works of Levinas and his notion of the ‘Other’ (Becker, 2013). In adopting such a perspective, the actions that we take, as individuals and organizations, are considered not merely by their utilitarian cost–benefit but also by their wider and further-reaching impact. A central element to this view is that the moral obligations of the individual (or organization) are not predicated upon a reciprocal arrangement, since “our obligations to others do not arise through entering into contracts with them to protect our mutual interests” (Soares, 2008, p. 546). Levinas emphasizes the literal and figurative ‘face’ that represents the ‘Other’ and the presence of which initiates a moral imperative to take responsibility for their wellbeing (Levinas, 1998).

The propinquity of ‘Others’ has been interpreted differently in the literature. For instance, Painter-Morland (2010) draws attention to the ethics of leadership that are concerned with the ‘Others’ within the corporation. Similarly, O’Leary (2015) recounts the breakdown in identification of, and responsibility for, the ‘Other’ that resulted in fraud within a stockbroking firm. These perspectives do not adequately reflect Levinas’ insistence upon our “*infinite responsibility*” for the ‘Other’ (Levinas, 1998, p. 124) but other literature does not place such bounds upon our responsibilities. This is an important distinction since true ‘responsible action’ requires the corporation to have “the liberty of taking the burden of the infinite responsibility for the Other—customer, employees, community, public at large” (Soares, 2008, p. 551). Andrade (2019) recognises the presence of ‘Others’ outside the organization through arguing that the benefits of financial aid programmes should be considered beyond their immediate ability to alleviate poverty. Desmond (2007) extends the notion of ‘Otherness’ to include humans and raw materials whose ‘face’ presents a moral imperative to be safeguarded, whilst Hatami and Firoozi (2018) usefully expand the notion of ‘Others’ to include the natural environment as a recipient, or even casualty, of the activities of organizations.

In addition, ‘infinite responsibility’ has generally been interpreted and applied spatially, across society and thereby maintains Levinas’ insistence upon the “face-to-face interaction with the Other” (Knights & O’Leary, 2006, p. 5). We acknowledge the views of Gladwyn et al. (1995) and Jonas (1984) and expand this interpretation temporally to also include responsibilities to future ‘Others’ and society.

Ultimately the Levinasian perspective considers our responsibility to others to be ‘infinite’ in extent (Bruna & Bazin, 2018). This has led some to challenge even the possibility of a Levinasian-based corporate ethics, describing the corporation as “*faceless*” (Bevan & Corvellec, 2007, p. 213), and to proffer that the moral compass of an organization is dependant upon the ethics of its human management that cannot be reduced to rules and legislation (Baker & Roberts, 2011; Faldetta, 2018; Mansell, 2008).

The notion of ‘the other’ is therefore of prime importance when discussing the pro-social acts of organizations. Whilst the majority of activities of an organization are concerned with its financial stability and sustainability, and many of its obligations toward society and the environment are legislated, some businesses perform acts that are not readily attributable to these norms. These acts are performed for the benefit of sometimes unknown ‘others’ without expectation of recognition or reward and often incurring substantial costs that are at odds with the fundamental business proposition.

## Pro-social Behaviours

A substantial corpus of literature has considered ‘pro-social behaviour’ as an inherent characteristic of humans who are thought to be naturally “*other-regarding*” (Jensen et al., 2014, p. 822; Silk & House, 2011). Studies have demonstrated these behaviours amongst both children and adults, but there is scepticism over what constitutes ‘authentic’ pro-social behaviour (Jensen, 2016).

Pro-sociality is often poorly defined but may be conceived of as a continuum of behaviours that span those that are demanded by legislation, those that are performed for clear and obvious gain, and those that are performed wholly for the benefit of others (Batson & Powell, 2003; PfattHeicher et al., 2021). It is the role of reciprocity that provides conceptual distinctiveness between types of non-mandated pro-social behaviours (Batson & Powell, 2003; Simpson & Willer, 2008). For instance, Simpson and Willer (2008, p. 50) insist upon reciprocity by maintaining “the potential for pro-sociality hinges on whether the act may be observed by others who will then bestow benefits on the helper at some later point in time”.

This is in stark contrast to the Levinasian notion of individual and corporate responsibility toward ‘Others’ that is not reliant upon reciprocity (Soares, 2008). Thus, a Levinasian approach would necessitate the adoption of ethical theoretical lenses that are independent of reciprocity. Supererogation comprises behaviours that are beyond an individual’s and an organization’s duties and moral obligations for which there is no expectation of reciprocity (Heyd, 1982). It therefore affords a means of examining the ‘good deeds’ of organizations that transcend those that are performed for material gain and are not readily explained by non-Levinasian ethical theory.

## Supererogation

Heyd’s (1982) seminal work provides a taxonomy of six supererogatory acts that comprise Moral Heroism, Beneficence, Volunteering, Favour, Forgiveness and Forbearance. These are acts that are laudable and beneficial but which may not be expected to form part of an individual’s or an organization’s moral obligations. Heyd (1982) provides four necessary conditions that need to be met in order for an act to be supererogatory: (i) supererogatory acts are neither obligatory nor forbidden, (ii) whose omissions are not wrong, and do not deserve sanction or criticism, (iii) are morally good, both by virtue of their (intended) consequences and by virtue of their intrinsic value, (iv) are done voluntarily for the sake of someone else’s good, and are thus meritorious.

Such actions may be described as ‘good to do but not bad not to do’ (Chisolm, 1963; Urmson, 1958), or are “*good because* [they] *advance*[s] *behaviour out of love and altruistic impulses*” (Fernandez-Dols et al., 2010, p. 525), or because they form part of a professional code of ethics (Stoff et al., 2016). However, this perspective of supererogation is not universal and some have pointed out that extreme examples of such acts may also be considered foolhardy (Souryal & Diamond, 2001). Occasionally, the term is even used in an apparently anachronistic manner by calling for organizations to perform more supererogatory acts (Power et al., 2017).

### Motivation and Intention

The reasons for performing supererogatory acts are numerous, complex and interdependent, comprising both deontic and aretaic dimensions (Levy, 2015; Benn, 2014; Hogan & Timmons, 2010). One may be motivated by a feeling of concern for a known, specific individual or group, or for an anonymous ‘other’ (Heyd, 1992) and acts that are performed for the benefit of unknown others are considered to be “*of the purest kind*” compared to those that are for the benefit of known others (p. 199).

Distinction is made between the characteristics of individuals or agents and the acts that they perform, and Heyd (1982) insists “*supererogation is primarily an attribute of acts or actions*” (p. 115). Benn (2017) also highlights the difference between the performer and the act itself in determining whether acts are supererogatory. When considering the performer, their motivations are a foundational component of the judgement of whether they acted supererogatorily. However, when considering the act that been performed, the motivations of the performer are a separate issue that do not bear upon our judgement (Heyd, 1992); Archer (2013, p. 452) states “*the motivation that led to the act does not alter the moral evaluation of the act; it only alters our evaluation of the agent*”.

As Archer (2013) and Benn (2019) insist, it is the ‘intention’ to perform the act (for example, to save someone from drowning) rather than the ‘motivation’ (for example, to attract publicity) that is important. If an act is performed to obviate negative repercussions, or is performed for personal glory, then that act could not be considered supererogatory even if the act itself was meritorious (Benn, 2018; Horgan & Timmons, 2010). Whether the performance of a supererogatory or otherwise virtuous act is indicative of the moral compass of the performing individual remains a moot point (Archer & Ridge, 2015; Archer, 2016a, b; Levy, 2015).

It is therefore important to identify the object of analysis and to clarify whether judgement is being made of the performer or the act. In choosing to observe the act “the answer must lie in the explanation of in what way these acts go beyond those that ‘merely fulfil’ duties” (Benn, 2017, p. 24).

### Supererogatory Acts

Three of Heyd’s (1982) types of supererogatory acts, comprising Moral Heroism, Beneficence and Volunteering, are described and evidenced in Mazutis’ (2014) deontic framework. Heyd (1982) describes Moral Heroism as “overcoming natural fears, desires, and considerations of self-interest, and also great self-sacrifice” (p. 144), Beneficence as “the contribution of one’s material goods” (p146) and Volunteering as “the offering of one’s services” (p150).

The remaining three acts, comprising Favour, Forgiveness and Forbearance, discussed next, are not evidenced by Mazutis (2014) and are therefore excluded from her framework.

#### Favour

Favours are a universal component of human behaviour, consisting of small acts of kindness that require comparatively little personal sacrifice (Heyd, 1982). Despite their apparent universality they are highly differentiated between cultures (Gueguen et al., 2016). Such is the power and significance of favours in certain societies that they have become discrete concepts, including, for example, the favour-exchanges that occur within Chinese ‘Guanxi’ (Liu & Jia, 2020), Russian ‘Blat’ (Cook et al., 2018) and Arabic ‘Wasta’ (Ali & Weir, 2020).

Favour exchange can occur between family members or amongst social structures (Thams et al., 2013) and most commonly appear in nations where there are “*institutional voids, limited social and geographic mobility, and strong reciprocity norms*” (Teagarden & Schotter, 2013, p. 447). In the workplace for instance, Chinese supervisors may grant favours to engender a sense of trust (Jiang et al., 2013), but such favours are used far less often in developed economies than in emerging economies (Teagarden & Schotter, 2013).

The degree of reciprocity of favours is also a differentiating factor (Teagarden & Schotter, 2013; Thams et al., 2013). Yakubovich’s (2012) examination of the post-Soviet job market suggested that recruitment favours were commonplace amongst friends but were not expected to be reciprocated. However, where favours are returned, it is generally done so soon after the initial favour has been provided to alleviate the “negative psychological state of indebtedness” (p. 499).

#### Forgiveness

Acts of forgiveness, or mercy and pardon, are not necessarily supererogatory. For instance, Heyd (1982) draws attention to the inappropriately harsh results of the application of a poorly formed law. Forgiveness is supererogatory when it is enacted in situations where the prescribed punishment would have been justified. There is a cultural dimension to forgiveness that reflects that of favours (Neto et al., 2007; Watkins et al., 2011), but it is also determined by other factors such as religiosity and emotional intelligence (Tsarenko & Tojib, 2012), empathy (Xu et al., 2012), and communication frequency and quality (Cehajic et al., 2008). It is even possible to measure degrees of forgiveness (Taysi & Orcan, 2017).

Being forgiven by others has a profound effect upon one’s own perspective on affording forgiveness to others in the future (Shevlin et al., 2017; Wohl & Branscombe, 2009). This is moderated by the degree of harm that one has received (Bombay et al., 2013) and the strength of prevailing social connections (Bapna et al., 2017). Fundamentally, forgiveness is governed by a complex web of personal and social factors that means that the act of forgiving and being forgiven does not necessarily result in the restoration of harmony (Palanski, 2012).

Ultimately, forgiving and forgiveness are necessary for mental wellbeing (Gromet & Okimoto, 2014; Lee et al., 2016; Myers et al., 2009). Mechanisms of forgiving may include decisional forgiveness where negative reprisals are eschewed in the hope of restoring relations, and emotional forgiveness where in accord with Tsarenko and Tojib (2015), negative emotions are replaced with positive thoughts (Watkins et al., 2011).

The act of forgiveness is one that is argued to be central to the development of a more just society (Blanco, 2016; Lacey & Pickard, 2015), but is also recognised to be something that is not without limits (Blanco, 2016). In a business context, where the study of forgiveness is a relatively recent development (Faldetta, 2021), apologies for brand misconduct are better received than defence (Tsarenko & Tojib, 2015), as are timely replies (Ghosh, 2017). Kurzynski (1998) advocates the development of ‘forgiveness as a virtue’ of managers, not only for its personal and social benefits but also because it is a reasonable response to the fact of life that one has to work with others “*with and without their faults*” (p. 82). Caldwell and Dixon (2010) even assert that forgiveness is a necessary characteristic of a successful business leader, but “*asking for forgiveness in a business context may be a relatively rare phenomenon*” (Goodstein et al., 2016, p. 33).

#### Forbearance

Acts of forbearance are primarily concerned with the ‘omission’ of actions that are entitled, such as the right of a creditor to demand payment. These acts “*involve doing less of the due amount of something which is undesirable to another person*” (Heyd, 1982, p. 153). Forbearance is rarely mentioned in the literature and where it is present the discussions are limited to its financial and economic roots. For instance, Lee et al. (2005) discuss the consequences of banks failing to retain sufficient capital to meet their obligations but are allowed to continue to operate and are thereby the recipients of ‘capital forbearance’ (Chang & Yu, 2017). Interestingly, and highlighting the non-financial repercussions of such an event, they state “*Forbearance causes a failing bank to adopt risk-shifting portfolio strategies, a situation known in the literature as moral hazard*” (p. 224). Markoczy et al. (2009) make an important observation that people of different backgrounds view forbearance and pro-social acts differently.

The bridge between what may at first be interpreted as ‘purely financial issues’ and what are ‘moral issues’ is further evidenced by Guth et al. (2015) who identify the conduct of ‘mutual forbearance’. This ensues, for example, when groups of organizations agree to avoid engaging in price wars or eschew developing competing products to prevent confrontation. Between the organizations this may be viewed as an act of morally sound forbearance. However, the virtue of such acts must also account for the perspective of the end-users or customers (one of the groups of ‘Others’ that are referred to by Soares (2008) or ‘faceless Others’ according to Levinas) of those organizations’ products and services who may be negatively impacted by what are ostensibly cartels.

## Supererogation Studies

Mazutis (2014) utilises the notion of supererogation to make headway in reconciling the tensions between duty and moral obligation through the development of a deontic framework that incorporates both the legislated and moral requirements of corporate actions. She distinguishes between those CSR actions that are mandatory and those that are voluntary, but which wider society may expect to be a part of an organization’s moral obligations.

Mazutis (2014) evidences Moral Heroism through the actions of Johnson & Johnson’s action to recall Tylenol after the capsules were found to contain cyanide and resulted in the deaths of several people (the capsules were laced with cyanide after despatch). Johnson & Johnson was credited for its role in undertaking a mass-media warning campaign that helped to address the problem but placed the organization in a financial predicament. A further example is provided in the case of Malden Mills, which continued to pay its workers whilst the factory was rebuilt after a fire. Beneficence is demonstrated in the charitable actions of corporations, along with Merck’s decision to provide medical aid to cure river blindness for free, and by organizations that give away medical solutions or providing charitable donations. Volunteering is illustrated in the actions of Tomasso Corporation by way of supporting employees whom had made redundant, and examples of organizations supporting other following natural disasters. Through the development of an ‘extended deontological framework’ that incorporates supererogation, this work made progress in explaining the activities of ‘rational commercial organizations’ that transcend expected boundaries and go beyond CSR and positive deviance.

Tencati et al. (2020) further advance our understanding of supererogation through focussing upon the need to differentiate between qualified and unqualified forms of supererogatory acts. Unqualified acts are motivated by personal choice, are altruistic and philanthropic, whereas qualified acts are those that are performed despite the presence of conditions that may excuse the act, for example, if performing the act would place the agent in mortal danger. Qualified supererogatory acts therefore consist of “*at least two levels of consideration*” (p. 260) that comprise the ‘first order’ reason for the action to be performed and a ‘second order’ justification for the act not to be performed.

They contend that the three organizational examples put forward by Mazutis’ (2014) can be interpreted within a wider context. For instance, they proffer that Johnson & Johnson’s decision to recall Tylenol “may have been strategic and financially motivated” (Tencati et al., 2020, p.  257) and the act therefore fails Heyd’s (1982) condition (iv) that it was done voluntarily for the sake of someone else’s good, and is thus meritorious. Consequently, they that argue that Mazutis’ (2014) “unqualified view of supererogation, in which the act absolutely goes beyond the call of duty, may have no real application in firms” (p. 258).

In advocating the superior value of qualified acts of supererogation, Tencati et al. (2020) advance the example of General Motors’ decision to pay the full annual bonus to its employees despite a shortfall in the company’s profits due to an expensive recall of defective components. From this they develop three conditions that are necessary for the acts of organizations to be considered supererogatory; (1) The action is other-regarding and brings significant benefits to stakeholders other than shareholders, (2) there are moral or utilitarian reasons strong enough to give the firm permission not to act, (3) there is not a clear business case for the firm.

### Critique of Supererogation Studies

#### Secondary Sources Only

First, both Mazutis (2014) and Tencati et al. (2020) rely upon secondary sources of information such as newspaper reports (e.g. Tencati et al.’s source for the General Motors example) or the “well-known story of Malden Mills” (Mazutis, 2014, p. 525). Whilst these have proved useful in evidencing the nature of supererogatory acts that organizations may perform, we suggest that there is now a need to acquire primary data to explore the intricacies of supererogatory theory. In addition, there is a need to explore the performance of supererogatory acts from within the firm and from a perspective that is ontologically and epistemologically closer to both those that are performing those acts and, importantly, those that are the recipients of those acts.

#### Singular Acts of Good

Second, Mazutis’ (2014) and Tencati et al.’s (2020) use of singular acts of ‘good deeds’ to evidence supererogation may be critiqued through infinite regression or extrapolation to identify some potentially valuable ‘business case’ for them to be performed and thereby render them null. Just as Tencati et al. (2020) critiqued the examples that are provided by Mazutis (2014), so can Tencati et al.’s (2020) examples be viewed within the wider sociohistorical context. For instance, Mazutis’ (2014) example of Johnson & Johnson recalling Tylenol can be interpreted as either a supererogatory act that potentially jeopardised the firm’s financial wellbeing, or, as Tencati et al. (2020) observe, was simply managers “protecting the Tylenol brand” (p. 257). Similarly, Tencati et al.’s (2020) discussion of General Motors’ payment of an annual bonus can be considered to be a morally laudable act that was financially burdensome, or, as Tencati et al. (2020) themselves postulate, was an act that also provided some benefit to the company by aiding “stable and constructive” (p. 260) relationships with the employees.

Levinas (1985, p. 80) stressed that the ‘face’ of the ‘Other’ is “meaningless without context” and Tencati et al. (2020) point out, what may have once been deemed to be supererogatory can become the norm over time. People inhabit multiple ‘holons’ (Gladwin et al., 1995), in which they may be ‘customers’, ‘employees’, ‘community’ or ‘public at large’ in several different social spaces, or even all of these at once (Card, 2005). Consequently, this results in tensions between what the organization is duty-bound to perform, what it is morally obligated to do, the duty and obligations of its staff (Rawwas et al., 2013) and what the wider society (of today and the future) judges its moral obligations to be.

For instance, General Motors’ decision was taken during a time of considerable upheaval due to the global financial crisis, and the United States Government bailout of the company was contingent upon radical restructuring (The White House, 2021). The media had challenged the appointment of General Motors’ new CEO, Mary Barra, who approved the payout of the bonus, as being based upon her identity as a successful, working mother (Arthur Page Society, 2015). If one were to adopt the perspective of the Government then the payment of the full bonus may be considered to be unnecessary. Contrastingly, the employees and the media largely viewed it as a laudable act. It could also be considered to be a necessary act to maintain consistency with the company’s new culture and image that was being widely and publicly communicated through a new social media campaign (Arthur Page Society, 2015). In order to exemplify the complexity in interpreting the moral value of this act, the ‘average American’ may have viewed the act as either laudable because it was beneficial for the company employees, simply as a means of currying the favour of the trade unions ahead of negotiations that were due to take place at the end of the year (Vlasic, 2015), or even as a waste of American taxpayers’ money. Tencati et al. (2020) highlight this challenge when they state that the decision by General Motors may be supererogatory “if these second-order reasons are strong enough” (p. 261).

Interpreting laudable acts is therefore contingent upon the observer’s own moral compass, their relationship to the organization, the stakeholders and the beneficiaries, not only at the time that the act was performed, but also within the wider socio-historical context. Even seemingly commendable initiatives can be found to be disturbingly lacking when examined in detail (Prasad & Elmes, 2005). We therefore maintain that by evidencing multiple instances and types of supererogatory acts within a single SME organization, this study is based upon an organization that has an authentic ‘different way of doing business’ and whose actions are not predicated upon commercial success, but are consistently ‘Other-regarding’.

#### Focus on Larger Companies

Third, in addition to the observation that the extant literature bases its examination of supererogation upon individual organizational examples, it is pertinent to note that these examples are also primarily concerned with the actions of larger, multinational organizations such as Johnson & Johnson (Mazutis, 2014) and General Motors (Tencati et al., 2020). Whilst larger organizations are undoubtedly important figureheads of responsible corporations, it must be recognized that the vast majority of global enterprises are SMEs: over 90% of global businesses are SMEs and employ 50% of the workforce (OECD, 2021; World Bank, 2021). Consequently, we argue that much of the change toward responsible management, and the achievement of many of the requirements that are encapsulated in the United Nation’s Sustainable Development Goals (United Nations, 2021), will be brought about by these smaller organizations since “given the economic and environmental significance of SMEs, they are important drivers of inclusive and green growth” (OECD, 2018, p. 4).

## Research Purpose

Heyd (1982) asserts that his taxonomy of supererogatory acts “*is not exhaustive nor its items mutually exclusive*” (p. 142). Whilst Mazutis (2014) has made significant headway in evidencing three of Heyd’s taxonomy of six acts, notwithstanding the issues that we have raised in our research questions above, the remainder of Heyd’s taxonomy has not yet been empirically evidenced. There is therefore a continued need to provide contemporary evidence of each type of act that is contained within Heyd’s taxonomy.

In building upon the works of Tencati et al. (2020) and Mazutis (2014) to address the research questions of this study, we turn to Forest Green Rovers football club, an SME that is globally recognized as being at the vanguard of social and environmental innovation both within its sector and across related domains. Through extended access to the organization, this research generates primary evidence of other-regarding supererogatory acts taken from the perspective of both the performant and the recipient.

### Research Context

Football is much more than just a popular sporting activity. It is the world’s leading sporting movement (Nielson, 2018; Schyns et al., 2016; Arau’jo et al., 2014) and is a part of the very fabric of some societies (Bal et al., 2011; Slack & Shrives, 2008). There are around 40,000 association football clubs in the UK, the oldest of which was founded in 1857. The English Premier League is the richest in the world, comprising the top twenty association football clubs, contributed £7.6 billion to the UK economy in the 2016–2017 season (Hughes & Ziegler, 2019) and employs around 100,000 people (Premier League, 2020).

The commercial importance of the game is significant and means that it “displays all the symptoms of inequality, short-termism and greed” (Lee, 1998, p. 32) and it is therefore under pressure to improve its accountability (Balmer, 2001; Brunzell & Söderman, 2012). Culturally, there is some evidence that football is moving away from its ultra-masculine, adversarial and profit-centred roots (Kolyperas et al., 2015) in order to “maximise its long-term positive impact on society, whilst simultaneously minimising its negative effect” (Anagnostopoulos & Shilbury, 2013, p. 268). Clubs have commenced engagement with the wider social and environmental actors and issues (Megheirkouni et al., 2018; Rouvrais-Charron & Kim, 2009) and develop CSR initiatives (Fifka & Jaeger, 2020). Whilst the results of this are laudable, there remains some doubt over the motivations to perform such acts (Blumrodt et al., 2013; Kolyperas et al., 2015) since they also result in improvements in brand awareness, sales, increased sponsorship and ultimately improved financial performance (Baena, 2018; Lee & Yoon, 2018; Nyadzayo et al., 2016; Sung & Lee, 2016). Whatever the fundamental reasons for these changes may be, they will require the drive and direction of new organizational actors to become fruitful endeavours for the sport and the wider social and ecological environs in which it operates (Anagnostopoulos & Shilbury, 2013).

#### The World’s Greenest Football Club Forest Green Rovers

Forest Green Rovers Football Club (FGR) was established in 1889 (Barnard, 2006) and one hundred and twenty-eight years later, in 2017, the club was promoted to Division Two in the English Football League and subsequently turned into a professional organization. However, the global media attention that has since followed has had very little to do with the club’s footballing prowess. Instead, the media, the general public, footballing governing bodies, organizations that share eco-conscious values, third party certificating organizations, other sporting organizations and even football aficionados have been attracted to the organization’s novel sustainability initiatives.

Whilst FGR’s use of solar panel charged floodlights, recycled water systems, energy-saving heating systems and charging points for electric cars may seem normal business practice, we posit that transferring these to football is radical in itself. More profound changes in the organization’s practices indicate a paradigmatic shift in the running of a professional football club (FGR Footprint Report, 2019). For instance, all staff (including the professional football players) and visitors (fans and dignitaries) are served vegan food only. FGR also developed the world’s first vegan playing surface (the pitch), match day tickets are carbon offset, family and community values are championed and recent eco-innovations have led to the player’s kit being made from bamboo fibre.

Subsequently, recognition for these achievements has been global. In 2017, the sport’s governing body (FIFA) labelled FGR as ‘the worlds greenest football club’ (BBC, 2018). In 2018 The United Nations certified FGR as *‘the world’s first carbon–neutral football club’* whilst also appointing Dale Vince (FGR’s Chairman) as a UN Climate Champion (The Guardian, 2018). In addition, the club has also claimed the mantle of being world’s first accredited vegan football club **(**Vegan Society, 2015**)**.

The success of FGR’s sustainability initiatives is argued to be a ‘big winner’ (FT, 2019) giving the organization a ‘competitive edge’ (The Sustainability Report, 2019). Financially, in 2018 the club reported a turnover of £4,921,685 and an operating profit of £422,647, which is approximately 19% higher than other professional football clubs operating at the same level (FT, 2019).

Despite being an SME that employs 129 people, FGR has fan groups from more than 20 countries outside England, including Russia, Norway and Holland, and in 2019 the launch of its new bamboo kit sold to 16 different countries across the globe including Australia, South Korea and Malaysia in just 24 h (FGR Footprint Report, 2019). Its media growth is also significant. In 2018 alone, the club recorded 2,303 media articles published across the globe, whilst expanding its social media presence from 687 Facebook ‘likes’ in 2012 to 37,442 in 2018, and 873 Twitter followers in 2012 to 27,657 in 2018 (FGR Footprint Report, 2019).

By 2018 the club had reported a 16% reduction in their carbon emissions, a 44% reduction of individual spectator’s carbon footprint, a 40% reduction in energy use and a 57% reduction in water consumption (FGR Footprint Report, 2019). In 2019 the organization was granted planning permission to build the world’s first all-wooden 5,000 capacity ‘Eco Park’ stadium, a development that has been described as:

The ‘Eco Park’ is set to be ‘the greenest football stadium in the world’. (The Guardian, 2019)

In summary, FGR is chosen as the research site since their externally validated sustainability and ethical practices “*shows a genuine excellence in their behaviour, going beyond what is reasonably expected of them*” (Tencati et al., 2020, p. 253).

## Methodology

Interpretive study is most useful in exploring and expanding new theoretical insight into organizational practice (Ciulli & Boe-Lillegraven, 2020; Langley & Abdallah, 2011) and affords the means of understanding the viewpoints of different actors (Moraes et al., 2020; Spiggle, 1994). Studies of organizational and individual ethics have frequently adopted an interpretivist approach to gain understanding of how the complex environments are navigated by their members (Ciulli et al., 2020; Jamali, 2010; Kourula & Delalieux, 2016; Moraes et al., 2017, 2020; Patton, 2002). In addressing the research questions, this study places emphasis upon gaining first-hand understanding of the ‘Other-regarding’ acts that are performed by FGR (Levinas, 1998) and is therefore a “*good fit*” with an interpretive approach (Karakas et al., 2017, p. 733).

Case study method is used to gain in-depth information of a research site and enable its contextually accurate interpretation (Davies, 2009). Extended case study examinations of single organizations are frequently used to gain deep understanding of complex situations (Davies, 2009; Jiang et al., 2021; Lamberti & Lettieri, 2009) and have been used for the exploration of ethical behaviour in organizations (Brigley, 1995; Crane, 1999; Gumey & Humphreys, 2006; Jiang et al., 2021; Kourula & Delalieux, 2016; Lamberti & Lettieri, 2009). The method also enables the acquisition of data from multiple sources that allows the triangulation of findings (Goulding, 2001; Longoni & Cagliano, 2018).

Large scale, single method approaches, such as those employed by Constandt et al. (2019), Wall-Tweedie and Nguyen (2018), Walker et al. (2010) have proven to be successful when exploring business ethics in sport. Such approaches allow the researcher to maximize data collection opportunities (Wall-Tweedie & Nguyen, 2018) and to prioritize what needs to be established (Stathopoulou et al., 2021).

Given the scope, manifold perspectives, multiple narratives, an ethicist football club and unique research context, the research team wanted to present a holistic view of FGR’s practice and the complexity in that practice. Consequently, a pluralistic approach (Chamberlain et al., 2011), that is a combination of multiple different methods of qualitative data collection (Šerić & Ljubica, 2018; McLeod, 2018), was employed to capture real life (Öberseder et al., 2011) and ethical leadership (Frisch & Huppenbauer, 2014) within FGR.

In this study, an extended, multi-method qualitative case study design was employed (Dyer & Wilkins, 1991; Yin, 2003) using participant observations, interviews and focus groups to capture a comprehensive picture of the way in which attitudes and motivations develop over time (Spanjaard et al., 2014). This approach enabled the capture of primary data from those that are both performing the acts and those that are the recipients of the benefits of those acts. Confirmation of findings was afforded through instrumental triangulation between methods, data triangulation between participant groups, and investigator triangulation during the process of data analysis and interpretation (Eisenhardt, 1989; Hall & Rist, 1999).

### Data Collection Methods

The lead author negotiated access to the club after attending a ‘business breakfast morning’ to discuss business sustainability and meeting the Chairwoman of FGR. Following this, he met with FGR’s Director, Community Development Team and the Public Relations Officer to discuss the scope of the intended research project. Subsequent to gaining approval to conduct the research through their University’s Research Ethics Committee and FGR’s Board of Directors, the study commenced.

A multi-phase protocol was followed comprising attendance at football matches to observe the everyday experiences and perceptions of the club’s staff and supporters, semi-structured interviews with key internal and external stakeholders of FGR, focus groups with visiting fans, and participation in FGR-run events (detailed in Table 1). This real-time, deep immersion (Peattie & Samuel, 2018), allowed the inductive construction of the complexities of ‘life at FGR’ (Hemingway & Starkey, 2018). Twenty-four semi-structured interviews were arranged with key management, players and operational staff to gain an understanding of the club’s core mission and activities (Boiral et al., 2019). Thirty-seven informal interviews were also conducted ‘in the moment’ throughout the period of engagement with the organization to explore opportune moments with visiting fans and local stakeholders (Blumer, 1969; Carpiano, 2009; Kusenbach, 2003). In total, 32.5 h of interviews were captured, which generated transcripts comprising 76,889 words. Three semi-structured focus groups were conducted with UK and international fans, in order to garner a wider interpretation of the activities of FGR. This generated a further 2.4 h of recordings and comprised approximately 7,235 transcribed words. The lead author’s fieldnotes were transcribed and contributed a further 27,852 words of data.

**Table 1:** Detail of data sets and empirical material

Data sets	Empirical material used in data analysis
Home matches attended as an FGR supporter = 27	**2017–18 Season** Exeter 9th September 2017 Morecambe 28th October 2017 Swansea under 21 s 31st October 2017 Crew 18th November 2017 Port Vale 6th January 2018 Coventry 3rd February 2018 Notts County 10th March 2018 Chesterfield 21st April 2018 **2018–19 Season** Port Vale 8th September 2018 Crawley 22nd September 2018 Grimsby 22nd January 2019 Notts County 9th February 2019 Yeovil 16th February 2019 MK Dons 30th March 2019 Exeter 4th May 2019 Tranmere Rovers 13th May 2019 **2019–20 Season (Cut Short due to Covid-19)** Newport 31st August 2019 Crawley 5th October 2019 Scunthorpe 7th December 2019 Salford 18th January 2020 Walsall 8th February 2020 Port Vale 11th February 2020
Away Matches attended as an FGR supporter = 3	Swindon 13th January 2018 Swindon 2nd February 2019 Cheltenham 2nd November 2019
Invitations to Events at FGR = 7	Meeting FGR Director, Community Development Team and Public Relations officer, 4th December 2018 FGR Community Day, 19th March 2019 FGR Sustainability Tour, 19th March 2019 Chairman Matchday Invitation FGR v Southampton under 21 s, 8th September 2019 Head Of Community Development / Head of Academy Matchday Invitation FGR v Colchester, 14th September 2019 Ex-Chairman Matchday Invitation FGR V Southhampton Under 21 s, 8th October 2019 Student visit for Sustainability Tour and Match, 9th February 2019 Student visit for Sustainability Tour and Match, 11th February 2020
Invitations to Events outside FGR = 2	Ex-Chairman keynote Presentation at the ‘Race for Sustainability’ Conference in Cardiff University 3rd December 2019 Ex-Chairman ‘Public Values Lecture’ at Cardiff University Business School 24th January 2020
Interviews with FGR Workforce = 12	Community Link Officer 4th May 2019 Community Ambassador 4th May 2019 Community Ambassador 4th May 2019 First Team Player 19th March 2019 First Team Player 19th March 2019 Professional Academy Football Player 19th March 2019 Professional Academy Football player 19th March 2019 Assistant Ground Person 3rd September 2019 Assistant Ground Person 3rd September 2019 Match Day Volunteer 8th October 2019 Catering Staff 8th October 2019 Front of House 8th October 2019
Interviews with FGR Management / Board of Directors = 12	Board Member 4th May 2019 Board Member 13th May 2019 Ex Director 19th March 2019 Ex Director 13th May 2019 First Team Academy Coach 19th March 2019 Head of Community Development 13th May 2019 Head Grounds Person 8th September 2019 Security Staff 8th September 2019 Chairman 8th September 2019 Head of Academy 14th September 2019 First Team Physiotherapist 14th September 2019 Ex-Chairman 8th October 2019 First Team Captain 8th October 2019
Interviews with FGR Supporters = 20	Male Supporter (Season Ticket Holder) 8th September 2018 Male Supporter (Season Ticket Holder) 8th September 2018 Female Supporter (Season Ticket Holder) 8th September 2018 Female Supporter (Season Ticket Holder) 16th February 2019 Male Supporter (Season Ticket Holder) 13th May 2019 Male Supporter (Season Ticket Holder) 13th May 2019 Male Supporter (Season Ticket Holder) 13th May 2019 Female Supporter (Season Ticket Holder) 13th May 2019 Male Supporter 8th September 2018 Male Supporter 8th September 2018 Female Supporter 8th September 2018 Female Supporter 16th February 2019 Female Supporter 16th February 2019 Male Supporter 16th February 2019 Male Supporter 16th February 2019 Male Supporter 13th May 2019 Male Supporter 13th May 2019 Female Supporter 13th May 2019 Male Supporter January 2020 Male Supporter January 2020 Visitor Female 4th May 2019 Visitor Male 4th May 2019 Visitor Male 4th May 2019
Interviews with FGR’s Local Community = 17	Resident, 30th May 2019 Church Representative, 19th March 2019 Forest Green Community Centre, 30th May 2019 Further Education College Lecturer 19th, 19th March 2019 School Teacher, 19th March 2019 Librarian, 29th May 2019 Tourist Information Centre, 29th May 2019 Sports Journalist, 13th May 2019 Community Development Project Manager, 30th May 2019 Youth Worker, 30th May 2019 Local resident, 31st May 2019 Local resident, 31st May 2019 Librarian, 19th March 2019 Tourist Information Centre, 19th March 2019 Shop Keeper, 30th May 2019 Shop Worker, 30th May 2019 Hotel Worker, 31st May 2019
Focus Groups with UK Football Fans = 2	Focus Group 1: 31st October 2017: 6 Participants (4 male / 2 Female) Focus Group 2: 15th February 2018: 5 Participants (3 male 2 Female)
Focus Group with International Football Fans = 1	Focus Group 3: 18th February 2019: 5 Participants (4 male 1 Female)

### Analytical Methods

The analysis took place cyclically, with the fieldnotes and prior rounds of analysis being used to develop further lines of enquiry (Harris, 2007; Wright et al., 2017). Our broad interpretation of the data sets was initially guided by the six fundamental supererogatory acts that are described in the literature review (Eisenhardt, 1989). We first sought out and coded the statements and descriptions of the sustainability activities that the club undertook. Following this we examined and coded the reactions of individuals to these activities to understand how they were interpreted in the context of a commercial sporting organization and thereby determine which were considered ‘dutiful’, or which were ‘above and beyond duty’ and therefore supererogatory. Throughout the process, our interpretation of the data was continuously reviewed through discussion between the authors, and with the fieldwork participants. Finally, we organized the codes according to Heyd’s (1982) taxonomy and an emergent theme (see Table 2).

**Table 2:** Taxonomy and an emergent theme coding

Semi structured interview data	Focus group data	Ethnographic data
*It’s not a secret, I’m also keen to tell others. I go to a few seminars and this year I'm going to Portugal to share my work.* (FGR Groundsperson) **Initial Code: Sharing** knowledge and practice	*I decided to try and reduce my carbon footprint. FGR showed me it’s possible…but it is hard! Less meat is obviously a good start* **Initial Code: Sharing** knowledge/**Sharing** landscape	*To be in a football match sponsored by itseasy2bvegan, offered free advice, samples of vegan products and chitchat on how to become a vegan was so far removed from my normal experience when going to a football game* **Initial Code: Sharing** landscape
*I think maybe the organic pitch that’s won awards and other teams and clubs are looking to copy that. Rolling that out and sharing our secrets is something really cool and different to do.* **(**FGR Supporter**)** **Initial Code: Sharing** knowledge and practice	*It was a brilliant day out and I learned a lot from the different people I met, the groundsman gave me some really interesting insights into how to create a vegan pitch and I really felt like the fans I met were friendly and proud and happy to talk about the fact that they were a green club* **Initial Code: Sharing** knowledge**/Sharing** landscape	*It’s amazing to see how much shared information is available around the ground, at every turn there are information board outlining the sustainability practices in operation* **Initial Code: Sharing** landscape**/Sharing** practice
*Our Chefs also share their ideas with other chefs and the ripple effect of what we do translates quite often when we go to other clubs.* (FGR Ex-CEO) **Initial Code: Sharing** knowledge and practice	*When you hear everybody talking about the changes, they’re making it really does motivate you to rethink your own buying habits* **Initial Code: Sharing** knowledge**/Sharing** landscape	*They are sharing knowledge on food, travel, energy and even how to grow an organic pitch* **Initial Code: Sharing** landscape**/Sharing** practice
*A lot of clubs talk to us about how we manage our pitch so for example Aston Villa, St Georges Park and Wembley we have talked a lot to them, and we have shared what we have done here.* (FGR Management) **Initial Code: Sharing** knowledge and practice	*They make sustainability look easy and possible for everyone to do something about it* **Initial Code: Sharing** knowledge**/Sharing** landscape	

Instrumental triangulation was afforded, in the analysis of Moral Heroism for example, through the responses of the Community Development Project Manager that confirmed those of Focus Group 1. Similarly, in the analysis of Forgiveness, the interpretation of the Crewe Fans Chant, Christmas 2018 was informed and confirmed by the statements made by the FGR CEO. Throughout the analyses, the voices of multiple FGR Supporters were garnered to confirm that their responses reflected collectively held viewpoints, and this provided data triangulation. Researcher triangulation is exemplified by the identification and confirmation of the emergent theme of ‘Sharing’ shown in Table 3. Discussions of the analyses and themes were conducted iteratively until Researcher consensus was achieved (Phillips et al., 2017; Skards & Thorbjornsen, 2014; Pecchioni & Halone, 2012).

**Table 3:** Emergent theme triangulation

Data	Theme	Supererogatory act
Dale Vince criticised	Leadership (Dale Vince)	Moral Heroism
Dale Vince investment		
Innovative practices	Ecological conscience	
Vegan only		
Beyond football		
Fans mocked	Steadfast in the face of criticism	
Staff under pressure		
Community events	Supporting local to global	Beneficence
School events		
Outreach programmes		
Vulnerable groups		
Bee hotel	Ecological contribution	
Meadow		
Kings Aftershave	Backing support groups	
Good to be Vegan		
Not in it for themselves	Caring	
Supportive		
Fame Lab	Donating club resources	Volunteering
Local/ National and Global events		
Helping local initiatives	Favour exchange	Favour
Organising litter picking events		
Helping source solutions to local problems	Favour facilitator	
Utilising its network		
Extending manager’s contract	Forgiving of sporting achievement	Forgiveness
Appreciating the style of play over winning		
Away fans’ chants	Forgiving of ridicule	
Milk smuggling	Forgiving of transgressions	
Players eating burgers		
Playing surface (pitch) condition and place	Forgiving of club facilities	
Paying a player’s salary after sale	Financial	Forbearance
Waste minimisation	Ecological	
Plastic reduction		
Planting wild area		
Discussing pitch with other football clubs	Sharing knowledge and practice of pitch management and maintenance	Sharing
Discussing football pitch with other sporting bodies		
Discussed electric vehicle usage with other football clubs	Sharing knowledge and practice of car use and charging	
Chefs share ideas with other chefs	Sharing knowledge and practice of Vegan food	
Safe environment to sample	Sharing landscape	
Free food samples to visitors		
Sharing the club's initiatives locally, nationally, and internationally	Sharing the club’s story of their journey	
Eco-trail	A landscape for sharing ‘sustainability’ knowledge and practice	
A constant source of education		

## Findings

This section is structured according to the six classes of supererogatory acts that are defined by Heyd (1982). It first provides confirmatory evidence of the acts of Moral Heroism, Beneficence and Volunteering, before addressing those of Favour, Forgiveness and Forbearance. Following this, it introduces the emergent class of supererogatory act that is ‘Sharing’.

### Moral Heroism

It is often difficult to encounter distinctive evidence of Moral Heroism since one can always counter the act by postulating that the motivations for performing it are coloured by some other non-supererogatory reasons. For instance, Mazutis’ (2014) example of Johnson & Johnson’s recall of Tylenol could be interpreted as the organization undertaking such actions that would portray them in a better light to stakeholders (particularly shareholders) and to any judicial procedures that may ensue at a later date. However, in applying the conditions that are used to qualify supererogatory acts, FGR has clearly committed financial and material resources for ecological improvement that would not be expected of an organization that was ‘simply a football club’. For instance, considerable investment has been made, not only in the duties that may be expected of CSR, but also in the development of innovatory practices such as the vegan pitch, the vegan food and the transition toward a supportive and family friendly, educational environment:

“[Dale Vince] puts all the money in to be more eco-friendly.” (Community Development Project Manager)

“It was excellent to see the different kinds of technologies supporting the eco-friendly ways Forest Green Rovers conduct themselves, this has obviously come at some expense to the club. (Focus Group 1)

We also find that the investments in the sustainability of the club do not just come with financial penalties. Football clubs are not only dependent upon their successes and popularity amongst fans, but also with paying sponsors. In an overtly masculine sector that is dominated by confrontation between the teams on the pitch, amongst fans in the grounds and between clubs in the football leagues, FGR’s radical break with tradition has require substantial moral fortitude by all its staff and stakeholders:

“A lot of people having obviously been getting a fair bit of criticism about the Chairman.” (FGR Supporter)

“Any time you’re pushing a point of view that’s non-traditional as far as football’s concerned then you’re going to attract criticism from others.” (FGR Supporter)

Even the Club’s owner comes under criticizm: as Wolf (1982) points out, ‘moral saints’ are not necessarily attractive human characters. However, his and the club’s achievements are simultaneously greatly admired:

“A lot of people don’t like him because he comes across as preachy…

But for us he’s preaching about something good so like I don’t care that he’s doing it.” (FGR Supporter)

It is this commitment to, and investment in, the voluntary and transformational behaviours in the club and its stakeholders that sets FGR’s efforts apart from other initiatives that may be classified as duty-bound CSR. Not only is the financial commitment considerable, but also the emotional and psychological commitment of FGR staff, along with its fans and sponsors, are necessary. This differs from Mazutis’ (2014) and Heyd’s (1982) interpretation of the term Moral Heroism purely in terms of the financial impact of supererogatory acts and extends it to include its impact upon personal, social and ecological factors that are also essential for the successful sustainable operation of the organization.

### Beneficence

FGR is a regular contributor to local events and is supportive of its immediate community, all of which are costly:

“They engage with the community, with the schools.” (FGR Supporter).

“They gave us half price tickets because I'm a youth worker.” (Community Development Project Manager).

“I mean people around as well they're all nice, all caring. They all look out for you. They are not just in it for themselves.” (FGR Professional Academy Football Player)

FGR also provides substantial support to vulnerable groups and to other sustainability-conscious organizations that are not associated with either the local community or the sport of football. This includes hosting a game to educate people on the plight of refugees, and allowing activist groups such as the Extinction Rebellion and Local Community Groups to promote their climate change causes at games. FGR’s Community Team also helped to address loneliness by visiting local residents who live on their own and providing them with vegan meals during the Covid-19 lockdown.

What is most apparent within the data are the club’s multi-faceted commitment to its purpose as a source of entertainment and sporting success, and also its ‘higher’ mission of taking responsible actions for the ecology. FGR supports small businesses such as the male aftershave social enterprise ‘Kings’ that supports mental health in young males, and ‘Good to be a Vegan’ who provide educational advice on the ecology and personal benefits of veganism. The club also allocates sections of their land to green spaces, wild eco meadows and bee hotels:

“We have a wild meadow and bee hotels at the entrance to our ground, that’s pretty cool. It makes you think, stop and do it yourself because you’re involved in it with the football.” (FGR Supporter)

This clearly demonstrates FGR’s supererogatory acts toward ‘Others’ outside the realm of football, and beyond its local community. To use our extension of Soares (2008) terminology and in accord with Hatami and Firoozi (2018) and Jonas (1984), the organization is committing its supererogatory actions toward known ‘Others’ and to future, unknown ‘Others’. Those ‘Others’ also include the future environment. The club’s sustainability initiatives and influence therefore transcend the real, notional and temporal boundaries of what may be expected of it as a ‘commercial sports organization’.

### Volunteering

FGR’s willingness to help others for no intrinsic reward is evident in their actions and their facilitation of others’ actions. FGR regularly offers the use of their organization’s resources to support the development of ecological practices, and promote wellbeing and community development in the local area. For instance, staff frequently volunteer to share their knowledge and understanding of ecological issues and impacts at local and national events:

“I’ve done this thing called Fame Lab at the Cheltenham Science Festival and I talked about how a vegan, vegetarian or piscetarian diet can help the environment.” (FGR Ambassador).

“I’ve followed the club through all the changes. I think it’s had an impact on me being surrounded by it as a fan, but obviously more so in my voluntary capacity and then in my employed role.” (FGR Community Link Officer).

There are clear similarities between FGR’s acts that may be classed as either beneficence or volunteering: as Heyd (1982) stressed, the classes of supererogatory acts are not mutually exclusive. We have taken the view that beneficent acts are those that incur some pecuniary deficit, whereas voluntary acts are those that make demands upon non-financial resources such as time. The voluntary execution of activities is also evident amongst the discussion of favours and favour-exchange that follows.

### Favour

There is a great deal of favour-exchange between FGR and its immediate network of community stakeholders. These are largely socially motivated favours that occur between the club and community interest groups, but also between individuals in the club and in the community. Some of these appear to have an unwritten expectation of reciprocity that has developed over time:

“The other week I was working with a resident who wanted to do a community litter-pick, and Sarah at FGR helped. We just help each other out that way.” (Local Community).

Unusually in the world of football, where confrontation and competition abound, the club has expended a tremendous amount of effort in sharing much of its newfound knowledge and expertise with other football teams. The vast majority of these favours are ecologically motivated and are performed for the benefit of the benefit of ‘Others’ within football but also ‘Others’ in different sporting environs:

“A lot of clubs talk to us about how we manage our pitch so Aston Villa, St Georges park, Wembley we have talked to a lot of them and we have shared what we have done here.” (FGR Ex-CEO).

“Wembley, Wimbledon, Sky TV and even Real Bettis from Spain have all been here, and they took a lot of ideas away I’ve also talked to the ground’s manager at St Georges Park and Wembley about the things I do here.” (FGR Groundsperson).

“We had someone from the UN here a few weeks ago and that speaks volumes really.” (FGR 1^st^ Team Player).

We also find that an intriguing aspect of favour-exchange is seen in FGR’s role as a ‘favour-facilitator’:

“A lot of work I have done here has gone beyond football. Most of my time has been connecting people up who can make sustainability.” (FGR Ex-CEO).

“If we work with a resident who expresses a concern, then we have contacts there that we can use to resolve it.” (Local Community).

The club has strong relationships with its immediate and wider communities (local and distant ‘Others’) and it uses these connections to enable favour-exchange to flourish between these ‘Others’. For instance, the local community requested a bin for discarded plastic bags to be placed at the local supermarket. A member of the FGR Community Team broached this with the club who forwarded the request to their waste management company, Grundon, who then addressed the problem for the community. Similarly, FGR host ‘community days’ whereby local community groups are hosted at the club’s ground and make use of their facilities. This is a beneficent act by the club, but their outreach activities with the local schools also means that:

I liked the fact that they got the local school involved in preparing the day.” (Local Community).

The literature on favours clearly indicates the important role of reciprocity in different cultures (Thams et al., 2013). However, contrary to much that has been written, within the FGR environ we find a substantial proportion of the favours that are performed are done so without an expectation of reciprocal actions. In fact, FGR plays a role as a broker of favours; such is its enduring relationships with the local community and its network of contacts in various sectors such as waste management (Thams et al., 2013). Further to this, the club’s willingness to share its approaches to ecological issues with ‘Others’ in the sport of football and, to reuse our earlier phrase, ‘beyond the boundaries of what may be expected of it as a commercial sports organization’, indicates a deep seated commitment to entrenching socially and ecologically sustainable attitudes and behaviours for the benefit of society at large.

### Forgiveness

Forgiving is not something that can be readily expected from or found within organizations (Caldwell & Dixon, 2010; Goodstein et al., 2016). We find multiple dimensions of forgiveness with the realm of FGR’s operations. First, and most surprising for a competitive sporting organization, the fans are forgiving of their team’s sporting performance and ameliorate any shortcomings by considering them in light of the club’s ‘greater performances’:

“Maybe they don’t have the best players to play like that and to win trophies because obviously it’s a small team. It’s not all about winning, it’s about putting a good performance and playing a certain style.” (FGR Supporter).

“Forest Green Rovers whether they win or lose on the pitch, they always win off it.” (Focus Group 2).

Similarly, whilst there has been substantial mockery of FGR’s vegan philosophy and practices, continued taunts are met with acceptance and good humour:

“Obviously, there’s still an element of away fans that want to chomp a sausage roll and chant ‘you can stick your vegan pie up your arse’ or whatever, but that’s football for you.” (FGR CEO).

“[singing to the tune of Band Aid’s ‘Feed The World’] *Feed the vegans let them know it's Christmas time.”* (Crewe Fans Chant, Christmas 2018).

The fans are also forgiving of those that transgress the club’s vegan principles and practices. Forgiveness applies to the club’s fans and also to the players:

“I was offered a sachet of ‘real’ milk from a home fan last week, who said he couldn't stand this vegan stuff. I did laugh, I call him ‘the milk smuggler’!” (FGR Supporter).

“I mean we all laugh when the players come out of the training ground and then they go straight down to the takeaway and get a McDonalds burger.” (FGR Supporter).

The club’s pursuit of sustainability has not been without its problems. For instance, maintaining the playing pitch without recourse to artificial treatments is challenging. However, the staff and supporters are mindful of those constraints, along with the peculiarities of the club’s physical location, but again are aware of the ultimate goal that is being worked toward:

“Obviously the organic pitch, it’s not been brilliant this season. But we are the highest ground in the country so I think that is part of the reason as well.” (FGR Board Member).

Contrary to the literature, and our own expectations, there was substantial evidence of occurrences of forgiving in and around FGR. In particular, the acts of forgiveness toward the club’s struggles with on-field success were unusual in this context. In addition, forgiveness for those that strayed from the club’s vegan philosophy was not something that we expected to observe. We infer that these instances were viewed as minor transgressions when viewed in light of the other considerable efforts that the club and its staff make toward a sustainable future (Bombay et al., 2013). This may be due to the family friendly culture that the club has engendered (Neto et al., 2007; Watkins et al., 2011). Through these acts of forgiveness the fans are able to ameliorate any negativity and, particularly through humour, replace them with positive thoughts and feelings (Watkins et al., 2011).

### Forbearance

Supererogatory acts of forbearance are, by definition, associated with the deferment of financial repayment (Chang & Yu, 2017; Lee et al., 2005). Within FGR we see some form of this in the club’s continued payment of a particular player’s salary, even though he had been placed on loan to another club who should have been paying his wages in line with custom and practice:

“The club was paying for Christian Doiche even when he was at Bolton.” (FGR Supporter).

There is little indication in the literature of how forbearance may be conceptualized in non-financial terms. We suggest that our study of FGR indicates a novel way in which forbearance can manifest in terms of the value that can be extracted from the environment. Like many other contemporary organizations, the club is committed to reducing its impact on the environment:

“We don’t use clingfilm at all at home now and we use refillable water bottles.” (FGR Community Link Officer).

However, the club’s activities are not merely concerned with limiting its current consumption of resources, but they also focus attention on reconstituting the environment:

“Forest Green Rovers are giving back some value to the ecosystem as opposed to exploiting it they see sustainability as a responsibility to preserving the planet and future generations to come. It’s inspiring to see these actions in such an industry.” (Focus Group 1).

This is also evident in the club’s construction of wild eco-meadows and bee hotels (discussed in Beneficence) and its support of other socially and ecologically concerned organizations.

We propose that these acts can be perceived of as ecological-forbearance. That is, the reduction of resource consumption may, in some instances, be perceived by ‘Others’ as a supererogatory act. We recognize that for the vast majority of organizations, particularly in developed economies, this is unlikely to be regarded ‘supererogatory’ since energy and carbon reduction initiatives have become ‘de rigueur’ and cost positive. However, in developing nations, and perhaps in some industries and sectors, acts of resource reduction that are not stipulated by legislation may be regarded as supererogatory. By extension, acts of ‘environmental reconstitution’ may be more likely to be regarded as supererogatory by current and future ‘Others’.

### ‘Sharing’

Throughout our analyses, we identified a recurrent theme that centred upon FGR’s willingness to share their knowledge and understanding with ‘Others’. This is clearly evident in the groundsman’s discussions with other sporting organizations (Favours), the support of small businesses that aim to educate people about sustainability (Beneficence), and the substantial work done by the FGR Ambassador programme (Volunteering). On reviewing the data we were struck by the frequency with which the club’s stakeholders, at all levels, used the term ‘share’ in their discussions (emphasis added):

“It’s not a secret, I’m also keen to tell others. I go to a few seminars and this year I'm going to Portugal to share my work.” (FGR Groundsperson).

“I think maybe the organic pitch that’s won awards and other teams and clubs are looking to copy that. Rolling that out and sharing our secrets is something really cool and different to do.” (FGR Supporte).

“We have done some sharing around electric vehicles and even Manchester United made some contact early on.

Our Chefs also share their ideas with other chefs and the ripple effect of what we do translates quite often when we go to other clubs. I remember when we got into League Two our very first game away was at Mansfield and they presented us with a huge vegan spread. That started to get repeated wherever we went.” (FGR Ex-CEO).

“We play good football but the most significant thing we do is share our story. We are the best club for showing what’s possible.” (FGR Ex CEO).

At a fundamental level, sharing of knowledge and understanding occurs through the provision of numerous information boards and an ‘Eco Trail’ around the ground. Whilst this may not be perceived to be a particularly novel feature, within the context of a football stadium it is radically different to what is usually experienced.

The primary means of sharing knowledge and focussing people’s attention upon their environmental impact and providing them with the practical means of reducing that impact was undertaken through their vegan-only ethos. FGR has become ‘famous’ as the ‘world’s first accredited vegan football club’, with players and fans being provided with only vegan food:

“Coming here introduced me to another world. Things like veganism and the carbon footprint of the stadium.” (FGR 1^st^ Team Captain).

Much of the education is provided at substantial cost to the club, such as through giving away vegan food samples, and giving talks and advice. One could perceive the vegan-only football environment as one that is driven by a vegan ideology. However, this does not appear to be the way that the vast majority of visitors interpret it. In contrast, whilst the data consistently highlights that ‘the food is good’, the message that a vegan diet is ‘good for the environment' is more important and also appears to be a view that has been adopted by many. Importantly, they view the club as a ‘safe space’ in which it is possible to explore vegan food and sustainable practices:

“I think our awareness of things like vegan food and other things like energy has been helped by the fact that we see it here every week here. We definitely eat less meat these days and are far more conscious of recycling.” (FGR Supporter).

“If you come here you can have a listen and have a learn.” (FGR 1st Team Captain).

Its efforts appear effective, and are being rewarded in material changes in the behaviours of its fans and local stakeholders:

“Forest Green is educating a lot of the youngsters.” (Youth Worker).

“You always get children coming back and saying, oh I recycle this now or I remembered to do that.” (FGR Head of Community Development).

“I’m not a vegan, but I certain eat a lot less meat than I used to.” (FGR Board Member).

## Discussion

### The Environment as ‘Other’

In addressing our research questions this study examines a single SME organization in which there is substantial evidence of the performance of multiple types of supererogatory acts. This identifies that such acts are not confined to larger organizations and adds credence to the assertion that the acts that have been observed are authentically ‘Other-regarding’ and supererogatory.

It also revealed substantial evidence of the performance of each of Heyd’s (1982) six classes of supererogatory acts within FGR. This is the first study to substantiate these ethical practices in organizations through the use of primary data and draws extensively upon the views of those that are involved in the performance and ‘Others’ that are in receipt of those beneficial acts. The acquisition of primary data also highlighted the importance of considering supererogatory acts toward non-human ‘Others’ (the environment) and afforded the means of identifying a new class of supererogatory actions that is ‘Sharing’ that extends Heyd’s (1982) taxonomy.

Mazutis (2014) and Heyd (1982) refer exclusively to acts of supererogation between people who share some common connection. This study found numerous instances of these acts that are extended toward diverse ‘Others’ who do not share a common connection. It also evidences acts that are performed for the benefit of the non-human ‘Other’ that is the current and future ecology. For instance, FGR’s owner and staff demonstrate their Moral Heroism in resisting criticism whilst endeavouring to implement their ecologically motivated initiatives. Similarly, the club’s other supererogatory acts all display a concern for the current and future environment, in addition to the individuals that are directly and indirectly involved with them.

Ezzamel and Willmott (2014) question the validity of the theoretical and practical divide that adopts either an anthropocentric or an ecocentric perspective, neither of which they argue is sufficient to achieve sustainability. They venture that organization and management theory as a whole is dominated by anthropocentric views of the world that “*impede the emergence of alternative (e.g. ecocentric) organisation theory*” (p. 1031). We highlight the importance of recognizing that supererogatory acts not only occur between individuals and organizations, but can also be directed toward the wider and future environment (non-human ‘Others’). This is an important observation since “*few people address human obligations regarding the nonhuman world*” (Gladwin et al., 1995, p. 879) and reinforces the need to consider all stakeholders “*including the natural environment*” (Tencati, et al., 2020, p. 250; Hatami & Firoozi, 2018).

### New Class of Supererogatory Act: Sharing

Applying the Heyd (1982) and Mazutis (2014) conditions for what constitutes supererogatory acts: (i) the sharing of knowledge and expertise is ‘neither obligatory nor forbidden’, (ii) the omission of sharing would not be wrong and would not deserve sanction or criticism, (iii) sharing is morally good, and (iv) sharing is done voluntarily for someone else’s good. Similarly, applying Tencati et al.’s (2020) three conditions for what constitutes supererogatory acts of organizations: (1) sharing is Other-regarding and brings significant benefits to stakeholders other than shareholders (it is done for the benefit of the wider and future ecology), (2) there are moral and utilitarian reasons that are, in our view, strong enough to give the firm permission not to act (they are demanding upon the financial and human resources which could otherwise be diverted to the club’s sporting success), (3) these acts of sharing have no clear business case for the firm (whilst they are important components of the club’s virtuous mission they are in opposition with its primary purpose as a sporting business). According to Heyd (1982), Mazutis (2014) and Tencati et al. (2020), these sharing activities therefore meet the conditions to be deemed supererogatory.

One can argue that ‘sharing knowledge and expertise’ comprises elements of beneficence and volunteering, which, as Heyd (1982) highlights, classes of supererogatory acts are not mutually exclusive. We therefore suggest that these activities form a class of supererogatory acts that are substantially different to those of Moral Heroism, Beneficence, Volunteering, Favours, Forgiveness and Forbearance. This claim is consistent with other work that has argued that ‘profit-*sharing*’ is a form of supererogatory act (Cortez, 2017). Importantly, our view of sharing extends the notion of supererogatory acts beyond those that are concerned with purely financial matters or are directed toward local ‘Others’. We maintain that the activities of FGR represent acts of sharing that are provided for the benefit of a wider community of ‘Others’ beyond the geographic local and beyond the arena of football. These acts are intended for the benefit of current and future ‘Others’ including the environment as a stakeholder. Acts are performed without expectation of reciprocation, or in the case of future ‘Others’ and the ‘environment as Other’, without even the possibility of reciprocation.

### Practical Implications

It is not uncommon for CSR and other pro-social endeavours to be tarnished with terms such as ‘greenwashing’, ‘image laundering’ or ‘woke-washing’. The perception of the authenticity of such initiatives appears to be contingent upon the degree to which multiple ‘good deeds’ are practiced and communicated to stakeholders and the wider realm of known and unknown, current and future ‘Others’. We posit that the taxonomy of supererogatory acts may afford a means for organizations to disaggregate their pro-social initiatives into multiple impactful acts. Collectively, these acts have the potential to convey conviction toward ‘doing good’ and obviate pejorative associations.

Additionally, an important distinction can be made between perceptions of the ‘true’ motivations of those that implement pro-social initiatives and the ultimate benefit that such initiatives deliver. As the literature clearly states, it is the intention of the act that is most important since the motivation behind it is difficult, if not impossible, to accurately convey. Consequently, managers should be mindful of the need to emphasize the benefits of their pro-social actions above the motivations to do so. Highlighting the improvements and experiences of the beneficiaries of such acts may help to focus stakeholder attention upon the results of the initiatives and diminish critical perceptions of the ‘true’ motivations. Organizations should be prepared for the scrutiny that may follow the performance and communication of acts that are deemed supererogatory, in particular, the negative feedback that may be experienced by individual managers and appropriate support mechanisms should be considered.

Finally, whilst supererogatory acts are laudable and may be a key feature of the pro-social initiatives of some organizations, and even of individual managers, it must be remembered that what is regarded as supererogatory today may become the expected norm tomorrow. Furthermore, supererogatory acts are context-dependant and may be interpreted differently across socio-spatial boundaries, cultures, nationalities and even periods of time. Organizations must therefore be mindful of the need to perform and communicate their supererogatory pro-social activities in ways that are contextually coherent.

## Conclusion

Supererogation has gradually gained interest as an ethical theory that explains those business practices that go above and beyond what is required by legislation and what may be expected by society. Whilst the limited extant literature has made valuable inroads into understanding such supererogatory acts, it is somewhat constrained by its use of singular instances of organizations performing such acts, a focus upon only large organizations, dependence upon secondary data sources and limited examples of the totality of forms that those acts may take.

This study addresses these issues through undertaking an extended examination of a single SME that is formally recognized for its work in addressing social and environmental issues at local, national and global levels. Primary data are captured through three years of participant observation, semi-structured interviews and focus groups, with participants that comprise those that are involved in performing the supererogatory acts and those that are the recipients of those benefits.

This study makes several important contributions in addresses the research questions.

First, it evidences a single organization that is engaged in the performance of multiple forms of supererogatory acts. This is important since it substantiates the claim that the acts that have been observed are authentically supererogatory and not merely ‘one-off’ acts of good nature. We propose that future study of supererogation within firms should seek to identify instances where multiple and/or sustained activities may be observed and evidenced.

Second, it confirms that supererogatory acts are not confined to large businesses that may have more ‘spare capacity’ to engage in such voluntary endeavours. SMEs are a significant component of economic and social activity and they form an important part of our collective efforts toward developing a sustainable future and the achievement of sustainable development goals. Research should seek to explore the manifestation of supererogation within firms of all types and size with views to uncovering the principles that motivate such activities and identify the factors that enable and constrain their performance.

Third, this is the first study to empirically substantiate the six types of supererogatory acts that are described in Heyd’s (1982) taxonomy. In doing so, it confirms Heyd’s observations that the classes of supererogatory acts are not mutually exclusive, and there is often considerable overlap between acts, such as those that are found between Beneficence and Volunteering. Research needs to be mindful of the ways in which these acts may be defined and interpreted and how they may differ across cultural norms. The research also extends Heyd’s taxonomy through the identification of a seventh class of supererogatory act that is ‘Sharing’. Acts of sharing are found to be directed toward local ‘others’ that are immediately connected with the organization, distant others who have no immediate connection with the organization, and to future others, including the environment, that may benefit from the organization’s initiatives. Future research should not disregard the non-human beneficiaries of such ‘good deeds’, which the literature claims is often overlooked.

## Limitations

This study was undertaken in one SME operating the sporting sector. Whilst this afforded the means of examining radical departures from the ethical norms of the industry, the findings would benefit from confirmation within other industries and organizational forms.

## Future Research

The introduction of the 'Sharing' class of supererogatory acts needs to be confirmed, whilst further primary evidence is needed to reveal the origins of an organization’s motivations to perform such acts.

Supererogation has emerged from Judeo-Christian concepts of morality and justice and it would therefore be useful to explore similar concepts and practices of things that are ‘good to do but not bad not to do’ that exist within other cultural contexts.

## References

[CR1] Ali S, Weir D (2020). Wasta: Advancing a holistic model to bridge the micromacro divide. Management and Organisation Review.

[CR2] Anagnostopoulos C, Shilbury D (2013). Implementing corporate social responsibility in English football: Towards multi-theoretical integration. Sport, Business and Management: An International Journal.

[CR3] Andrade J (2019). They Can Be Choosers: Aid, Levinas and unconditional cash transfers. African Journal of Business Ethics.

[CR4] Araújo N, de Carlos P, Antonio Fraiz J (2014). Top European football clubs and social networks: a true 2.0 relationship?. Sport, Business and Management.

[CR5] Archer A (2013). Supererogation and intentions of the agent. Philosophia.

[CR6] Archer A (2016). Motivational judgement internalism and the problem of supererogation. Journal of Philosophical Research.

[CR7] Archer A (2016). Are acts of supererogation always praiseworthy?. Theoria.

[CR8] Archer A, Ridge M (2015). The Heroism Paradox: Another paradox of supererogation. Philosophy Studies.

[CR9] Arthur Page Society. (2015). Retrieved from https://page.org/attachments/1edd5184509636aabbdc07ef0c3546e0facf5c0c/store/00f8d9b37cec4d725c881b7235467635aeab21b4d2333398c819839d426b/General-Motors-Case-Study-2015.pdf

[CR10] Baena V (2018). The importance of CSR practices carried out by sport teams and its influence on brand love: The Real Madrid Foundation. Social Responsibility Journal.

[CR11] Baker M, Roberts J (2011). All in the Mind? Ethical identity and the allure of corporate responsibility. Journal of Business Ethics.

[CR12] Bal C, Quester P, Plewa C (2010). Emotions and sponsorship: A key to global effectiveness? A comparative study of Australia and France. Asia Pacific Journal of Marketing and Logistics.

[CR13] Balmer JMT (2001). The three virtues and seven deadly sins of corporate branding. Journal of General Management.

[CR14] Bapna R, Qiu L, Rice S (2017). Repeated interactions versus social ties: Quantifying the economic value of trust, forgiveness, and reputation using a field experiment. Misquarterly.

[CR15] Barnard T (2006). Something to Shout About: The History of Forest Green Rovers FC.

[CR16] Barnett, M. L., Henriques, I., & Husted, B. W. (2020). Beyond Good Intentions: designing CSR initiatives for greater social impact. *Journal of Management*, forthcoming.

[CR17] Batson, C. D., & Powell, A. A. (2003). Altruism and prosocial behaviour. In Millon, T., & Lerner, M. J. (Eds.), *Handbook of psychology*. Wiley

[CR18] BBC. (2018). Forest Green Rovers Named ‘Greenest Football Club in World’. Retrieved from https://www.bbc.co.uk/news/uk-england-gloucestershire45677536.

[CR19] Becker PA (2013). The contribution of Emmanuel Levinas to corporate social responsibility and business ethics in the post-modern era. Journal of International Business Ethics.

[CR20] Benn C (2014). What is wrong with promising to supererogate. Philosophia.

[CR21] Benn C (2017). Supererogation, optionality and cost. Philosophical Studies.

[CR22] Benn C (2018). The Enemy of the Good: Supererogation and requiring perfection. Utilitas.

[CR23] Benn C (2019). Intentions, motives and supererogation. The Journal of Value Inquiry.

[CR24] Bevan D, Corvellec H (2007). The impossibility of corporate ethics: For a Levinasian approach to managerial ethics. Business Ethics: A European Review.

[CR25] Blanco A (2016). Fair compensation with different social concerns for forgiveness. Review of Economic Design.

[CR26] Blumer H (1969). Symbolic interactionsim: Perspective and method.

[CR27] Blumrodt J, Desbordes M, Bodin D (2013). Professional football clubs and corporate social responsibility. Sport, Business and Management.

[CR28] Boiral O, Ebrahimi M, Kuyken K, Talbot D (2019). Greening remote SMEs: The case of small regional airports. The Journal of Business Ethics.

[CR29] Bombay A, Mathesson K, Anisman H (2013). Expectations among aboriginal people in Canada regarding the potential impacts of a government apology. Political Psychology.

[CR30] Brigley S (1995). Business ethics in context: Researching with case studies. Journal of Business Ethics.

[CR31] Bruna MG, Bazin Y (2018). Answering Levinas’ call in organization studies. European Management Review.

[CR32] Brunzell T, Söderman S (2012). Board evaluation in the top Nordic football clubs. Sport, Business and Management.

[CR33] Caldwell C, Dixon RD (2010). Love, forgiveness, and trust: Critical values of the modern leader. Journal of Business Ethics.

[CR34] Card RF (2005). Individual responsibility within organisational contexts. Journal of Business Ethics.

[CR35] Carpiano RM (2009). Come take a walk with me: The “go-along” interview as a novel method for studying the implications of place for health and well-being. Health and Place.

[CR36] Carroll AB (1979). A three-dimensional conceptual model of corporate social performance. Academy of Management Review.

[CR37] Cehajic S, Brown R, Castano E (2008). Forgive and forget? Antecedents and consequences of intergroup forgiveness in Bosnia and Herzegovina. Political Psychology.

[CR38] Chamberlain K, Cain T, Sheridan J, Dupuis A (2011). Pluralisms in qualitative research: From multiple methods to integrated methods. Qualitative Research in Psychology.

[CR39] Chang C, Yu M (2017). Valuing vulnerable mortgage insurance under capital forbearance. Journal of Real Estate Finance Economics.

[CR40] Chisholm RM (1963). Supererogation and offence: A conceptual scheme for ethics. Ratio.

[CR41] Ciulli F, Kolk A, Boe-Lillegraven S (2020). Circularity brokers: Digital platfrom organizations and waste recovery in food supply chains. Journal of Business Ethics.

[CR42] Constandt B, De Waegeneer E, Willem A (2019). Ethical code effectiveness in football clubs: A longitudinal analysis. Journal of Business Ethics.

[CR43] Cook, C. E, Murphy, L., & Thomas, B. (2018). An evolutionary framework exploring the role of periodisations in the modern development of a Baltic State: the case of HRM in the Latvian Public Sector. *Economic and Industrial Democracy*.

[CR44] Cortez FGF (2017). Employee profit sharing: A moral obligation or a moral option?. Kritike.

[CR45] Crane A (1999). Are you ethical? Please tick yes or no: On researching ethics in business organizations. Journal of Business Ethics.

[CR46] Davies IA (2009). Alliances and networks: Creating success in the UK Fair Trade Market. Journal of Business Ethics.

[CR47] Desmond J (2007). Levinas: Beyond egoism in marketing and management. Business Ethics: A European Review.

[CR48] Dyer WG, Wilkins AL (1991). Better stories not better constructs, to generate better theory: A rejoinder to Eisenhardt. Academy of Management Review.

[CR49] Eisenhardt KM (1989). Building theories from case study research. Academy of Management Review.

[CR50] Ezzamel M, Willmott H (2014). Registering ‘the Ethical’ in organisational theory formation: Towards the disclosure of an ‘Invisible Force’. Organisation Studies.

[CR51] Faldetta G (2018). A relational approach to responsibility in organizations: The logic of gift and Levinasian ethics for a ‘corporeal’ responsibility. Culture and Organization.

[CR52] Faldetta, G. (2021). Forgiving the Unforgivable: the possibility of the ‘unconditional’ forgiveness in the workplace. *Journal of Business Ethics*.

[CR53] Fernandez JL, Camacho J (2016). Effective elements to establish an ethical infrastructure: An exploratory study of SMEs in the Madrid region. Journal of Business Ethics.

[CR54] Fernandez-Dols J-M, Aguilar P, Campo S, Vallacher RR, Janowsky A, Rabbia H, Brussino S, Lerner MJ (2010). Hypocrites or maligned cooperative participants? Experimenter indiced normative conflict in zero sum situations. Journal of Experimental Social Psychology.

[CR55] FGR Footprint Report. (2019). *Forest Green Rovers to Compensate Carbon Emissions Related to Fan Travel.* Retrieved from https://sustainabilityreport.com/2019/07/31/forestgreen-rovers-to-compensate-carbon-emissions-related-to-fan-travel/.

[CR56] Frisch C, Huppenbauer M (2014). New insights into ethical leadership: A qualitative investigation of the experiences of executive ethical leaders. Journal of Business Ethics.

[CR57] FT. (2019). Green Credentials Prove Big Winner for Tiny English Football Team. https://www.ft.com/content/d66ba036-763a-11e9-be7d-6d846537acab.

[CR58] Fukuda K, Ouchida Y (2020). Corporate Social Responsibility (CSR) and the Environment: Does CSR increase emissions?. Energy Economics.

[CR59] Ghosh T (2017). Managing Negative Reviews: The persuasive role of webcare characteristics. Journal of Internet Commerce.

[CR60] Gladwin TN, Kennelly JJ, Krause T-S (1995). Shifting paradigms for sustainable development: Implications for management theory and research. Academy of Management Review.

[CR61] Goodstein J, Butterfield K, Neale N (2016). Moral repair in the workplace: A qualitative investigation and inductive model. Journal of Business Ethics.

[CR62] Gosselt JF, Thomas R, Haske L (2019). Won’t get fooled again: The effects of internal and external (CSR EC)-labeling. Journal of Business Ethics.

[CR63] Goulding C (2001). Grounded theory: A magical formula or a potential nightmare. Marketing Review.

[CR64] Gromet DM, Okimoto TG (2014). Back Into The Fold: The influence of offender amends and victim forgiveness on peer reintegration. Business Ethics Quarterly.

[CR65] The Guardian. (2019). *Forest Green Rovers Granted Planning Permission for All Wooden Stadium*. Retrieved from https://www.theguardian.com/football/2019/dec/29/forest-green-rovers-granted-planning-permission-for-all-wooden-stadium

[CR66] Gueguen N, Meineri S, Ruiz C, Pascual A (2016). Promising Reciprocity: When proposing a favor for a request increases compliance even if the favor is not accepted. The Journal of Social Psychology.

[CR67] Gumey PM, Humphreys M (2006). Consuming responsibility: The search for value at Laskarina Holidays. Journal of Business Ethics.

[CR68] Guth W, Hager K, Kirchkamp O, Schwalbach J (2015). Testing forbearance experimentally: Duopolistic competition of conglomerate firms. International Journal of the Economics of Business.

[CR69] Hall AL, Rist RC (1999). Integrating Multiple Qualitative Research Methods (or avoiding the precariousness of a one-legged stool). Psychology & Marketing.

[CR70] Harris N (2007). Corporate engagement in processes for planetary sustainability: Understanding corporate capacity in the non-renewable resources extractive sector, Australia. Business Strategy and the Environment.

[CR71] Hatami A, Firoozi N (2017). A dynamic stakeholder model: An other-oriented ethical approach. Business Ethics: A European Review.

[CR72] Hemingway CA, Starkey K (2018). A falling of the veils: Turning points and momentous turning points in leadership and the creation of CSR. Journal of Business Ethics.

[CR73] Heyd D (1982). Supererogation.

[CR74] Heyd D (1992). Experimentation on trial why should one take part in medical research?. Bioethics and the Law.

[CR75] Hira A (2020). The Hollow Core: Breakdowns in global governance of CSR.

[CR76] Horgan T, Timmons M (2010). Untying a Knot from the Inside out: Reflections on the “paradox” of supererogation. Social Philosophy & Policy.

[CR77] Hughes, M., & Ziegler, M. (2019). *Premier League’s £7.6bn Contribution to UK Economy*. Retrieved from https://www.thetimes.co.uk/article/premier-leagues-7-6bn-contribution-to-uk-economy-glrz53vf6.

[CR78] Humphreys M, Brown AD (2008). An analysis of corporate social responsibility at credit line: A narrative approach. Journal of Business Ethics.

[CR79] Jamali D (2010). The CSR of MNC subsidiaries in developing countries: Global, local, substantive or diluted?. Journal of Business Ethics.

[CR80] Jensen K (2016). Prosociality. Current Biology.

[CR81] Jensen K, Vaish A, Schmidt MFH (2014). The Emergence of Human Prosociality: Aligning with others through feelings, concerns, and norms. Frontiers in Psychology.

[CR82] Jiang X, Chen CC, Shi K (2013). Favor in Exchange for Trust? The role of subordinates’ attribution of supervisory favors. Asia Pacific Journal of Management.

[CR83] Jiang, X., Prokopovych, B., & DiStefano, G., (2021). Leveraging A Lenient category in Practicing Responsible Leadership A Case Study. *Journal of Business Ethics*, (In Print).

[CR84] Jonas H (1984). The imperative of responsibility: In search of an ethics for the technological age.

[CR85] Karakas F, Sarigollu E, Uygur S (2017). Exploring the diversity of virtues through the lens of moral imagination: A qualitative inquiry into organizational virtues in the Turkish context. Journal of Business Ethics.

[CR86] Knights D, O’Leary M (2006). The possibility of ethical leadership. Journal of Business Ethics.

[CR87] Kolyperas D, Morrow S, Sparks L (2015). Developing CSR in professional Football clubs: Drivers and phases. Corporate Governance.

[CR88] Kourula A, Delalieux G (2016). The Micro-level Foundations and Dynamics of Political Corporate Social Responsibility: Hegemony and Passive Revolution through Civil Society. Journal of Business Ethics.

[CR89] Kurzynski MJ (1998). The virtue of forgiveness as a human resource management strategy. Journal of Business Ethics.

[CR90] Kusenbach M (2003). Street phenomenology: The go-along as ethnographic research tool. Ethnography.

[CR91] Lacey N, Pickard H (2015). To blame or to forgive? Reconciling punishmentand forgiveness in criminal justice. Oxford Journal of Legal Studies.

[CR92] Lamberti L, Lettieri E (2009). CSR Practices and corporate strategy: Evidence from a longitudinal case study. Journal of Business Ethics.

[CR93] Langley A, Abdallah C, Bergh D, Ketchen D (2011). Templates and turns in qualitative studies of strategy and management. Building methodological bridges: Research methodology in strategy and management (6.

[CR94] Premier League. (2020). *Economic Impact*. Retrieved from: https://www.premierleague.com/this-ispl/the-premier-league/686502.

[CR95] Lee S, Brown A (1998). Grey shirts to grey suits. Fanatics, identity and fandom in football.

[CR96] Lee E, Yoon S (2018). The effect of customer citizenship in corporate social responsibility (CSR) activities on purchase intention: The important role of the CSR image. Social Responsibility Journal.

[CR97] Lee S, Lee J, Yu M (2005). Bank capital forbearance and valuation of deposit insurance. Canadian Journal of Administrative Sciences.

[CR98] Lee SS, Jung EJ, Kim J, Lee S (2016). Why does forgiving boost creativity? The role of cognitive persistence. Seoul Journal of Business.

[CR99] Levinas E (1998). Otherwise than being or beyond essence: Translated by Alphonso Lingis.

[CR100] Levinas, E. (1985) Éthique et Infini. Dialogues d’Emmanuel Levinas et Philippe Nemo. Paris: Fayard Ethics and infinity. Pittsburgh: Duquesne University Press.

[CR101] Levy DK (2015). Assimilating supererogation. Royal Institute of Philosophy Supplement.

[CR102] Liu, L., & Jia, Y. (2020). Guanxi HRM and Employee Well-being in China. *Employee Relations*.

[CR103] llia, L., Zyglidopoulos, S., Romenti, S., Rodrı ´guez-Ca ´novas, B. & Adel Valle Brena, A. (2013). Communicating Corporate Social Responsibility to a Cynical Public. *MIT Sloan Management Review*, 16–19.

[CR104] Longoni A, Cagliano R (2018). Sustainable innovativeness and the triple bottom line: The role of organizational time perspectives. Journal of Business Ethics.

[CR105] Mansell S (2008). Proximity and Rationalisation: The limits of a Levinasian ethics in the context of corporate governance and regulation. Journal of Business Ethics.

[CR106] Margolis JD, Walsh JP (2003). Misery loves companies: Rethinking social initiatives by business. Administrative Science Quarterly.

[CR107] Markoczy L, Vora D, Xin K (2009). Forbearance in organizational citizenship behaviour. The International Journal of Human Resource Management.

[CR108] Mazutis D (2014). Supererogation: Beyond positive deviance and corporate social responsibility. Journal of Business Ethics.

[CR109] McWilliams A, Siegel D (2001). Corporate social responsibility: A theory of the firm perspective. Academy of Management Review.

[CR110] Megheirkouni M, Amaugo A, Jallo S (2018). Transformational and transactional leadership and skills approach: Insights on stadium *management*. International Journal of Public Leadership.

[CR111] Moraes C, Carrigan M, Bosangit C, Ferreira C, McGrath M (2017). Understanding ethical luxury consumption through practice theories: A study of fine jewellery purchases. Journal of Business Ethics..

[CR112] Moraes C, Kerrigan F, McCann R (2020). Positive shock: A consumer ethical judgement perspective. Journal of Business Ethics.

[CR113] Myers E, Hewstone M, Cairns E (2009). Impact of conflict on mental health in Northern Ireland: The mediating role of intergroup forgiveness and collective guilt. Political Psychology.

[CR114] Neto F, Pinto C, Mullet E (2007). Seeking forgiveness in an intergroup context: Angolan, Guinean, Mozambican, and East Timorese Perspectives. Regulation & Governance.

[CR115] Nielson. (2018). *World football Report. *Retrieved from https://www.nielsen.com/uk/en/insights/report/2018/world-football-report/#.

[CR116] Nyadzayo MW, Leckie C, McDonald H (2016). CSR, relationship quality, loyalty and psychological connection in sports. Marketing Intelligence & Planning.

[CR117] O’Leary M (2015). Work identification and responsibility in moral breakdown. Business Ethics: A European Review.

[CR118] Öberseder M, Schlegelmilch BB, Gruber V (2011). “Why Don’t Consumers Care About CSR?”: A qualitative study exploring the role of CSR in consumption decisions. Journal of Business Ethics.

[CR119] OECD. (2018). SMEs: Key drivers of green and inclusive growth. Retrieved July 6, 2021, from https://www.oecd.org/greengrowth/GGSD_2018_SME%20Issue%20Paper_WEB.pdf.

[CR120] OECD. (2021). OECD and Entrepreneurship Outlook 2019. Retrieved from July 6, 2021, from https://www.oecd.org/industry/smes/SME-Outlook-Highlights-FINAL.pdf.

[CR121] Ohreen DE, Petry RA (2012). Imperfect duties and corporate philanthropy: A Kantian approach. Journal of Business Ethics.

[CR122] Okoye A (2009). Theorising corporate social responsibility as an essentially contested concept: Is a definition necessary?. Journal of Business Ethics.

[CR123] Painter-Morland M (2010). Questioning corporate codes of ethics. Business Ethics: A European Review.

[CR124] Palanski ME (2012). Forgiveness and Reconciliation in the Workplace: A multi-level perspective and research agenda. Journal of Business Ethics.

[CR125] Patton M (2002). Qualitative research & evaluation methods.

[CR126] Peattie K, Samuel A (2018). Fairtrade towns as unconventional networks of ethical activism. Journal of Business Ethics.

[CR127] Pecchioni LL, Halone KK (2000). Relational Listening II: Form & variation across social and personal relationships. International Journal of Listening.

[CR128] Pfattheicher S, Nielsen YA, Thielmann I (2021). Prosocial Behaviour and Altruism: A review of concepts and definitions. Current Opinion in Psychology.

[CR129] Phillips SR, Mercier K, Doolittle S (2017). Experiences of teacher evaluation systems on high school physical education programs. Physical Education and Sport Pedagogy.

[CR130] Power S, Di Domenico M, Miller G (2017). The nature of ethical entrepreneurship in tourism. Annals of Tourism Research.

[CR131] Prasad P, Elmes M (2005). In the Name of the Practical: Unearthing the hegemony of pragmatics in the discourse of environmental management. Journal of Management Studies.

[CR132] Rawwas YA, Arjoon S, Sidani Y (2013). An Introduction of Epistemology to Business Ethics: A study of marketing middle-managers. Journal of Business Ethics.

[CR133] Renard MC (2003). Fair trade: Quality, market and conventions. Journal of Rural Studies.

[CR134] Rouvrais-Charron C, Kim C (2009). European football under close scrutiny. International Journal of Sports Marketing and Sponsorship.

[CR135] Samuel A, Taylor D, White GRT, Norris M (2018). Unpacking the authenticity gap in corporate social responsibility: Lessons learned from Levi’s ‘Go Forth Braddock’ campaign. Journal of Brand Management.

[CR136] Sarker S, Searcy C (2016). Zeitgeist or chameleon? A quantitative analysis of CSR definitions. Journal of Cleaner Production.

[CR137] Schyns B, Gilmore S, Dietz G (2016). What lessons can we learn from football about leadership and management? Leadership lessons from compelling contexts. Emerald Group Publishing Limited.

[CR138] Shevlin T, Thornock J, Williams B (2017). An examination of firms’ responses to tax forgiveness. Review of Accounting Studies.

[CR139] Silk JB, House BR (2011). Evolutionary foundations of human prosocial sentiments. Proceedings of the National Academy of Sciences of the United States of America.

[CR140] Simpson B, Willer R (2008). Altruism and Indirect Reciprocity: The interaction of person and situation in prosocial behaviour. Social Psychology Quarterly.

[CR141] Skard S, Thorbjørnsen H (2014). Is publicity always better than advertising? The role of brand reputation in communicating corporate social responsibility. Journal of Business Ethics.

[CR142] Slack RE, Shrives PJ (2008). Social disclosure and legitimacy in Premier League football clubs: The first ten years. Journal of Applied Accounting Research.

[CR143] Soares C (2008). Corporate legal responsibility: A Levinasian perspective. Journal of Business Ethics.

[CR144] Souryal SS, Diamons DL (2001). The rhetoric of personal loyalty to superiors in criminal justice agencies. Journal of Criminal Justice.

[CR145] Spanjaard D, Young L, Freeman L (2014). Emotions in supermarket brand choice: A multimethod approach. Qualitative Market Research: An International Journal.

[CR146] Spiggle S (1994). Analysis and interpretation of qualitative data in consumer research. Journal of Consumer Research.

[CR147] Stathopoulou A, Quansah TK, Balabanis G (2021). The Blinding Effects of Team Identification on Sports Corruption: Cross-Cultural Evidence from Sub-Saharan African Countries. Journal of Business Ethics.

[CR148] Stoff BK, Scully K, Householder AL, Fabbro S, Kantor J (2016). The American Academy of Dermatology Pledge. Journal of the American Academy of Dermatology.

[CR149] Sung M, Lee W (2016). What makes an effective CSR program? An analysis of the constructs of a cause-related participant sport sponsorship event. International Journal of Sports Marketing and Sponsorship.

[CR150] Taysi E, Orcan F (2017). The conceptualization of forgiveness among Turkish children and adolescents. International Journal of Psychology.

[CR151] Teagarden MB, Schotter A (2013). Favor prevalence in emerging markets: A multi-level analysis. Asia Pacific Journal of Management.

[CR152] Tencati A, Misano N, Castaldo S (2020). A Qualified Account of Supererogation: Toward a better conceptualization of corporate social responsibility. Business Ethics Quarterly.

[CR153] Thams Y, Liu Y, Glinow MAV (2013). Asian Favours: More than a cookie cutter approach. Asia Pacific Journal of Management.

[CR154] The Sustainability Report. (2019). Has Sustainability Given Forest Green Rovers a Competitive Edge on the Pitch? Retrieved from https://www.sustainabilityreport.com/2019/08/19/has-sustainability-given-forest-green-rovers-a-competitive-edge-on-the-pitch/

[CR155] The White House. (2021). Retrieved from https://obamawhitehouse.archives.gov/realitycheck/the-press-office/fact-sheet-obama-administration-auto-restructuring-initiative-general-motors.

[CR156] Tsarenko Y, Tojib D (2012). The role of personality characteristics and service failure in consumer forgiveness and service outcomes. Journal of Marketing Management.

[CR157] Tsarenko Y, Tojib D (2015). Consumers’ Forgiveness after brand transgression: The effect of the firm’s corporate social responsibility and response. Journal of Marketing Management.

[CR158] United Nations. (2021). The 17 Goals. Retrieved from https://sdgs.un.org/goals

[CR159] Urmson JO, Melden AI (1958). Saints and heroes. Essays in moral philosophy.

[CR160] Vegan Society. (2015). *Green Football Club is First to Receive the Vegan Trademark.* Retrieved from: https://www.vegansociety.com/whats-new/news/green-football-club-firstreceive-vegan-trademark.

[CR161] Vlasic, B. (2015) Despite Recalls, G.M. Pays Workers a Big Bonus. Retrieved from: https://www.nytimes.com/2015/02/05/business/gm-reports-2-8-billion-profit-in-2014.html

[CR162] Vredenburg J, Kapitan S, Spray A, Kemper JA (2020). Brands taking a stand: Authentic brand activism or woke washing. Journal of Public Policy and Marketing.

[CR163] Walker M, Heere B, Parent MM, Drane D (2010). Social responsibility and the Olympic Games: The mediating role of consumer attributions. Journal of Business Ethics.

[CR164] Wall-Tweedie J, Nguyen SN (2018). Is the grass greener on the other side? A review of the Asia-Pacific sport industry’s environmental sustainability practices. Journal of Business Ethics.

[CR165] Watkins DA, Hui EKP, Luo W, Regmi M, Worthington EL, Hook JN, Davis DE (2011). Forgiveness and interpersonal relationships: A Nepalese investigation. The Journal of Social Psychology.

[CR166] White GRT, Wang X, Li D (2015). Inter-Organisational Green Packaging Design: A case study of influencing factors and constraints in the automotive supply chain. International Journal of Production Research.

[CR167] Wohl MJA, Branscombe NR (2009). Group threat, collective angst, and ingroup forgiveness for the war in Iraq. Political Psychology.

[CR168] Wolf S (1982). Moral saints. The Journal of Philosophy.

[CR169] World Bank. (2021). Small and Medium Enterprises (SMEs) Finances. Retrieved from: https://www.worldbank.org/en/topic/smefinance

[CR170] Wright AL, Zammuto RF, Liesch PW (2017). Maintaining the values of a profession: Institutional work and moral emotions in the emergency department. Academy of Management Journal.

[CR171] Xu H, Kou Y, Zhong N (2012). The effect of empathy on cooperation, forgiveness, and ‘Returning Good for Evil’ in the Prisoner’s Dilemma. Public Personnel Management.

[CR172] Yakubovich V (2012). Getting a job as a favor in the Russian Post-Socialist Labor Market. Asia Pacific Journal of Management.

[CR173] Yin RK (2003). Case study research: Design and methods.

